# Special Issue on Energy Harvesters and Self-Powered Sensors for Smart Electronics

**DOI:** 10.3390/mi12121455

**Published:** 2021-11-26

**Authors:** Qiongfeng Shi, Huicong Liu

**Affiliations:** 1 Department of Electrical and Computer Engineering, National University of Singapore, 4 Engineering Drive 3, Singapore 117583, Singapore; 2 School of Mechanical and Electric Engineering, Jiangsu Provincial Key Laboratory of Advanced Robotics, Soochow University, Suzhou 215021, China

In recent years, we have witnessed the revolutionary innovation and flourishing advancement of the Internet of things (IoT), which will maintain a strong momentum even more with the gradual rollout of the fifth generation (5G) wireless network and the rapid development of personal healthcare electronics. Enabled by the ultrahigh data communication rate of 5G technology, various IoT systems can be envisioned by linking numerous multifunctional electronic devices together in an integrated and interconnected network, such as in smart homes, smart buildings, industry 4.0, smart cities, unmanned shops, wearable body area networks, point-of-care diagnosis and treatment, etc. In these complicated systems with widely distributed device nodes, the energy supply in the IoT era is gradually migrating from the regularly replaced battery-based supply mode toward the sustainable in situ supply mode. Compared to batteries, energy harvesting technologies that scavenge available energies from the ambient surroundings exhibit great merits in supplying energy to IoT systems, e.g., highly extended and unlimited lifetime, great portability, flexible/stretchable compatibility, and sustainability. Therefore, various energy harvesting technologies and devices are undergoing rapid and significant innovation, providing key functionalities in diverse IoT systems as energy harvesters and/or self-powered sensors. Accordingly, this Special Issue showcases nine research articles that focus on the novel development of energy harvesters/self-powered sensors and their broad applications in IoT systems and smart electronics. The research articles in this Special Issue explore the following aspects of energy harvesting technologies such as electromagnetic energy harvesters, piezoelectric energy harvesters, and hybrid energy harvesters: mechanism design, structural optimization, performance improvement, and a wide range of energy harvesting and self-powered monitoring applications.

In terms of the electromagnetic energy harvesters, Li et al. [1] designed a magnetically coupled electromagnetic energy harvester composed of two spring-connected magnets, one free magnet and coils, for scavenging the low-frequency human motion energy. The main advantage of the magnetic-spring structure is the weakened repulsive force, enabling effective low-frequency energy harvesting. Under handshaking (0.2 g and 3.4 Hz) and leg movement excitation, the energy harvester obtained a maximum output voltage of 0.6 V and a maximum output power of 26 mW. Cheng et al. [2] investigated in detail the characteristics of a diamagnetically stabilized levitation structure by both simulation and experiment. They found out that the symmetric monostable levitation system is more stable, and further explored the optimal parameters, achieving an output voltage of 250.69 mV and output power of 86.8 μW. Huang et al. [3] also proposed a magnetic-coupled electromagnetic energy harvester with nonlinear features and a broad bandwidth through the strong coupling between an iron core and vibrating magnets. After optimization, the energy harvester realized a broad operation bandwidth of 17–30 Hz and a high output power of 174 mW (at 35 Ω) that was able to light up 360 parallel-connected LEDs.

As for the piezoelectric energy harvesters, Song et al. [4] studied the enhancement effect of two piezoelectric energy harvesters in the context of water flow-induced vibration. The performance correlation of the upstream and the downstream piezoelectric energy harvester was theoretically and experimentally investigated. Thereupon, optimization was performed to boost the total output power which was significantly higher than that of a single piezoelectric energy harvester. Huang et al. [5] proposed an automatic data-driven strategy to optimize the design of piezoelectric energy harvesters, based on the algorithm of generalized pattern search (GPS), which can effectively save designers’ effort and achieve more precise parameters. Employing the optimal length and thickness, the derived energy harvester showed an increment of 371% and 1000% in output power and normalized power density, respectively. Han et al. [6] developed a bimetallic-cantilever-triggered piezoelectric energy harvester for self-powered and automatic heat-event monitoring. If the temperature reaches a preset threshold, the bimetallic cantilever will detach from the piezoelectric energy harvester, causing the resonance and output generation of the piezoelectric energy harvester. Under continuous heating, such temperature-triggered cycles were repeated with electric power generation. Zhang et al. [7] investigated a rope-driven piezoelectric energy harvester for broadband and low-frequency energy harvesting, which consists of a low-frequency cantilever and a high-frequency cantilever connected by a rope. After systematic simulation and experiment, the characteristics of the rope-driven energy harvester were validated, indicating that its performance could be conveniently adjusted by the rope margin or stiffness. Huang et al. [8] presented a multi-DOF piezoelectric energy harvester with a wide bandwidth using the frequency interval shortening design. Configured with one straight cantilever and two U-shaped cantilevers with proof masses, the energy harvester exhibited five low resonances, i.e., 13, 15, 18, 21 and 24 Hz, constituting a broad operation bandwidth at low frequencies.

Based on the hybridized mechanism of electromagnetics and piezoelectricity, Li et al. [9] proposed an electromagnetic hybridized scheme to improve the output performance of a piezoelectric energy harvester. The mass block of a piezoelectric cantilever was replaced by an alternating magnet array and a coil array was added beside the magnet array, forming the hybrid energy harvester. It achieved a power density of 3.53 mW/cm^3^ at the acceleration of 0.3 g and frequency of 18.6 Hz, which is 686 times higher than that of a basic cantilever piezoelectric energy harvester. In addition, the hybrid energy harvester also exhibited excellent performance in charging capacitors or batteries, e.g., a 2.2 mF capacitor was charged up to 8 V within 17 s, showing great promise in developing self-powered electronics in IoT and wearable applications.

We would like to express our sincere appreciation to all the authors for submitting their papers to this Special Issue. Meanwhile, we would also like to thank all the reviewers for dedicating their valuable time and helping to ensure the quality of this Special Issue.
